# Integrative taxonomy of the rock-dwelling gecko *Cnemaspis siamensis* complex (Squamata, Gekkonidae) reveals a new species from Nakhon Si Thammarat Province, southern Thailand

**DOI:** 10.3897/zookeys.932.50602

**Published:** 2020-05-12

**Authors:** Natee Ampai, Perry L. Wood Jr, Bryan L. Stuart, Anchalee Aowphol

**Affiliations:** 1 Department of Zoology, Faculty of Science, Kasetsart University, Bangkok, 10900 Thailand Kasetsart University Bangkok Thailand; 2 Department of Biology, Faculty of Science, Srinakharinwirot University, Bangkok, 10110 Thailand Srinakharinwirot University Bangkok Thailand; 3 Department of Biological Sciences and Museum of Natural History, Auburn University, Auburn, AL, USA Auburn University Auburn United States of America; 4 Section of Research and Collections, North Carolina Museum of Natural Sciences, Raleigh, NC, USA North Carolina Museum of Natural Sciences Raleigh United States of America

**Keywords:** *
Cnemaspis
*, morphology, phylogeny, species diversity, taxonomy, Thailand

## Abstract

The rock-dwelling gecko genus *Cnemaspis* is one of the most species-diverse genera of gekkonid in Thailand. Earlier studies relied on morphological data to identify species, but cryptic morphology often obscured species diversity in *Cnemaspis*. In this study, an integrative taxonomic approach based on morphological characters and sequences of the mitochondrial NADH dehydrogenase subunit 2 (ND2) gene were used to clarify current taxonomy of the *Cnemaspis siamensis* complex and delimit a new species from Lan Saka District, Nakhon Si Thammarat Province, southern Thailand. *Cnemaspis lineatubercularis***sp. nov.** is distinguished from other congeneric species by the combination of morphological characters: (1) maximum snout-vent length (SVL) of 40.6 mm (mean 38.8 ± SD 1.4, *N* = 12) in adult males and maximum SVL of 41.8 mm (mean 39.5 ± SD 1.9, *N* = 7) in adult females; (2) 8–9 supralabial and infralabial scales; (3) gular, pectoral, abdominal, and subcaudal scales keeled; (4) rostral, interorbitals, supercilium, palmar scales, and ventral scales of brachia smooth; (5) 5–6 small, subconical spine-like tubercles present on flanks; (6) 19–21 paravertebral tubercles linearly arranged; (7) 27–29 subdigital lamellae under the fourth toe; (8) 4–7 pore-bearing precloacal scales, pores rounded arranged in chevron shape and separated only in males; (9) one postcloacal tubercles each side in males; (10) ventrolateral caudal tubercles present anteriorly; (11) caudal tubercles restricted to a single paravertebral row on each side; (12) single median row of subcaudal scales keeled and lacking enlarged median row; and (13) gular region, abdomen, limbs and subcaudal region yellowish only in males. Genetically, the uncorrected pairwise divergences between the new species and their congeners in the *C. siamensis* group were between 15.53–28.09%. The new species is currently known only from granitic rocky streams at Wang Mai Pak Waterfall in the Nakhon Si Thammarat mountain range. Its discovery suggests that additional unrecognized species of *Cnemaspis* may still occur in unexplored areas of southern Thailand.

## Introduction

The rock-dwelling gecko genus *Cnemaspis* Strauch, 1887 is one of the most speciose genera in the family Gekkonidae. The genus is geographically widespread from tropical Africa eastward through South Asia, southward to Southeast Asia (Bauer et al. 2007; Grismer et al. 2014). However, recent molecular phylogenetic analyses of *Cnemaspis* suggest the genus may be polyphyletic, with three separate, unrelated clades consisting of African, South Asian, and Southeast Asian clades (Gamble et al. 2012; Pyron et al. 2013).

Southeast Asian *Cnemaspis* is a monophyletic group (Gamble et al. 2012; Pyron et al. 2013) that contains 59 species distributed from Laos, southern Vietnam, Cambodia, Thailand, southward through the Thai-Malay Peninsula to Borneo, Java, and Sumatra (Bauer and Das 1998; Das 2005; Bauer et al. 2007; Grismer and Ngo 2007; Grismer et al. 2009, 2014; Grismer and Chan 2010; Kurita et al. 2017; Riyanto et al. 2017; Uetz et al. 2019). In Thailand, there are currently 17 recognized species of *Cnemaspis* (Grismer et al. 2010, 2014; Wood et al. 2017; Ampai et al. 2019; Uetz et al. 2019), ranging from Kanchanaburi Province, western Thailand (Grismer et al. 2010) to Chanthaburi Province, eastern Thailand (Bauer and Das 1998), southward through southern Thailand and its offshore islands (Grismer et al 2014; Wood et al. 2017; Ampai et al. 2019). Based on the combination of morphological characters and molecular data, Grismer et al. (2014) indicated that *Cnemaspis* species from Thailand belong to four species groups, consisting of the *affinis* group, the *chanthaburiensis* group, the *kumpoli* group (= Pattani clade of Grismer et al. 2014) and the *siamensis* group.

The *siamensis* group is the most species-diverse of the *Cnemaspis* group in Thailand, and the number of described species in the group has increased rapidly during the past decade (e.g., Grismer et al. 2010, 2014; Wood et al. 2017; Ampai et al. 2019). Currently, there are eleven recognized species in the *siamensis* group: *C. adangrawi*Ampai et al., 2019, *C. chanardi*Grismer et al., 2010, *C. huaseesom*Grismer et al., 2010, *C. kamolnorranathi*Grismer et al., 2010, *C. omari*Grismer et al., 2014, *C. phangngaensis*Wood et al., 2017, *C. punctatonuchalis*Grismer et al., 2010, *C. roticanai* Grismer & Onn, 2010, *C. siamensis* Smith, 1925, *C. thachanaensis*Wood et al., 2017 and *C. vandeventeri*Grismer et al., 2010. The *siamensis* group is diagnosed by having a moderate body size of 37.8–49.6 mm snout-vent length (SVL); 7–11 supralabials; 6–11 infralabials; 0–8 pore-bearing precloacal scales; 15–29 paravertebral tubercles; 1–3 postcloacal tubercles in males; 21–31 lamellae beneath the fourth toe; and a light-colored prescapular crescent (Grismer et al. 2010, 2014; Wood et al. 2017; Ampai et al. 2019). Despite the high number of species already described in the *siamensis* group in Thailand, additional taxonomic diversity likely exists (Grismer et al. 2010, 2014; Wood et al. 2017).

More than half of Southeast Asian *Cnemaspis* species have been described primarily or solely on the basis of morphological characteristics (Smith 1925; Taylor 1963; Bauer and Das 1998; Das and Leong 2004; Das 2005; Bauer et al. 2007; Grismer et al. 2010). However, morphological data alone has been insufficient for resolving some taxonomic issues that are confounded by morphological crypsis. During the past decade, an integrative taxonomic approach that uses multiple sources of data (e.g., morphology, DNA sequencing, ecology, biogeography, behavior) to delimit species and describe taxa (Dayrat 2005) has been shown to be very effective in revealing cryptic species diversity and microhabitat specialization in Southeast Asian *Cnemaspis* (e.g., Grismer et al. 2013, 2014; Wood et al. 2013, 2017; Kurita et al. 2017; Ampai et al. 2019).

During fieldwork in October 2016 and January 2019, we collected specimens of the *C. siamensis* group at Wang Mai Pak Waterfall, Lan Saka District, Nakhon Si Thammarat Province, southern Thailand that could not be referred to any named species. We examine qualitative and quantitative (univariate and multivariate analyses) variation in morphology and mitochondrial DNA sequence data and show that the Lan Saka specimens differ from all other species of *Cnemaspis*. On the basis of this integrative approach, we described the Lan Saka population as a new species.

## Materials and methods

### Sampling

Specimens of *Cnemaspis* were collected by hand during the day (1000–1800 h) and at night (1900–2200 h) between October 2016 and January 2019 from Wang Mai Pak Waterfall, Lan Saka District, Nakhon Si Thammarat Province, Thailand. Liver samples for genetic analysis were taken from euthanized specimens and preserved in 95% ethanol. Specimens were then fixed in 10% formalin and later transferred to 70% ethanol for permanent storage. Specimens and tissue samples were deposited in the herpetological collection at the Zoological Museum of Kasetsart University, Bangkok, Thailand (**ZMKU**) and the Thailand Natural History Museum, Pathum Thani, Thailand (**THNHM**). Comparative material was also examined in the holdings of these institutions (Appendix 1), and comparative data were obtained from the original descriptions of other *Cnemaspis* species in Thailand (Grismer et al. 2010; Wood et al. 2017; Ampai et al. 2019).

### Morphological measurements

The following morphometric measurements were taken by the first author on the left side of preserved specimens to the nearest 0.1 mm using digital calipers under a Nikon SMZ 745 dissecting microscope. Morphological measurements were taken only from adult individuals as determined by the presence of secondary sexual characteristics including the presence of hemipenes or pore-bearing precloacal scales in males, and the presence of calcium glands or eggs in females. Sixteen morphological measurements were taken following Grismer et al. (2014) and Wood et al. (2017): snout-vent length (**SVL**), taken from tip of snout to the anterior margin of vent; tail width (**TW**) at the base of the tail immediately posterior to the postcloacal swelling; tail length (**TL**), as distance from the vent to the tip of the tail, whether original or regenerated; forearm length **(FL**), taken on the dorsal surface from the posterior margin of the elbow while flexed 90° to the inflection of the flexed wrist; tibia length (**TBL**), taken on the ventral surface from the posterior surface of the knee while flexed 90° to the base of the heel; head length (**HL**), as distance from the posterior margin of the retroarticular process of the lower jaw to the tip of the snout; head width (**HW**) at the angle of the jaws; head depth (**HD**), as the maximum height of head from the occiput to the throat; axilla-groin length (**AG**), taken from the posterior margin of the forelimb at its insertion point on the body to the anterior margin of the hind limb at its insertion point on the body; eye diameter (**ED**), as the maximum horizontal diameter of the eyeball; eye-ear distance (**EE**), measured from the anterior margin of the ear opening to the posterior edge of the eyeball; ear length (**EL**), taken from the greatest vertical distance of the ear opening; eye-nostril distance (**EN**), measured from the anterior most margin of the eyeball to the posterior margin of the external nares; eye-snout distance (**ES**), measured from the anterior margin of the eyeball to the tip of snout; inner orbital distance (**IO**), as the width of the frontal bone at the level of the anterior edges of the orbit; and internarial distance (**IN**), measured between the medial margins of the nares across the rostrum.

Meristic characters of scales and qualitative observations of other structures were made through a Nikon SMZ 745 dissecting microscope. The external observations of meristic characters were taken following Grismer et al. (2014) and Wood et al. (2017): number of supralabial (**SUP**) and infralabial (**INF**) scales, counted from below the middle of the orbit to the rostral and mental scales, respectively; texture of scales on the anterior margin of the forearm; number of paravertebral tubercles (**PVT**) between limb insertions, counted in a straight line immediately left of the vertebral column; general size (i.e., strong, moderate, weak) and arrangement (i.e., random or linear) of dorsal body tubercles; number of subdigital lamellae beneath the fourth toe (= 4^th^ toe lamellae), counted from the base of the first phalanx to the claw; presence or absence of a row of enlarged, widely spaced, tubercles along the ventrolateral edge of the body flank between limb insertions; number, orientation and shape of pore-bearing precloacal scales; relative size of subcaudal and subtibial scales; and number of postcloacal tubercles on each side of tail base.

### Morphological analysis

Statistical analyses were used to compare differences in size and shape in the *siamensis* group, including the Lan Saka samples (*N* = 19) and four congeners in the *siamensis* group: *C. adangrawi* (*N* = 8), *C. chanardi* (*N* = 7), *C. omari* (*N* = 5) and *C. siamensis* (*N* = 8). Other species in the *siamensis* group (*C. huaseesom*, *C. kamolnorranathi*, *C. phangngaensis*, *C. punctatonuchalis*, *C. roticanai*, *C. thachanaensis*, and *C. vandeventeri*) were not included in the morphometric analyses due to lack of specimens. Five putative operational taxonomic units (OTUs) were assigned on the basis of observed variation in morphometric analysis. Fifteen morphometric variables (SVL, TW, FL, TBL, HL, HW, HD, AG, EE, ED, EL, EN, ES, IO, and IN) were corrected for differences in ontogenetic composition by the following allometric equation: X_adj_ = X – β(SVL – SVL_mean_), where X_adj_ is the adjusted value of the morphometric variable; X is the original value; β is the within-clade coefficient of the linear regression of each original character value (X) against SVL; SVL = snout-vent length; SVL_mean_ = overall average SVL length of OTUs (Thorpe 1975, 1983; Turan 1999; Lleonart et al. 2000). Tail length (TL) was not included due to the differences in length between original and regenerated tails. Univariate analyses were implemented in the statistic software PAST 3.24 (Hammer et al. 2001) using an analysis of variance (ANOVA) to compare morphological differentiation in traits among the Lan Saka samples and the five congeners in the *siamensis* group. ANOVAs having *p*-value less than 0.05 were subjected to a Tukey’s honestly significant difference (HSD) test to identify all pairwise comparisons among sample means for significant differences (*p* < 0.05).

Multivariate analyses were performed using the base statistical software in RStudio v. 1.2.1335 (RStudio Team 2018). A principal component analysis (PCA) using the built-in R functions: prcomp (R Core Team 2018) and ggplot2 (Wickham 2016) were performed to find the best low-dimensional space of morphological variation in data. Principal components (PCs) with eigenvalues greater than 1.0 were retained in accordance to the criterion of Kaiser (1960). A discriminant analysis of principal components (DAPC) was performed using the adegenet function (Jombart 2008) to characterize clustering and distance in morphospace. The DAPC was used for all congeners to find the linear combinations of morphological variables that have the greatest between-group variance and the smallest within-group variance. The DAPC relies on data transformation using PCA as a prior step to ensure that variables included in the discriminant analysis (DA) are uncorrected and number fewer than the sample size (Jombart et al. 2010).

### Genetic analysis

Genomic DNA was extracted from liver tissue of five individuals of *Cnemaspis* (Table 1) using the Qiagen DNAeasy tissue kit (Valencia, CA, USA). A 1,251 bp fragment of mitochondrial (mt) DNA consisting of the NADH dehydrogenase subunit 2 (ND2) gene and the flanking tRNAs Trp, Ala, Asn, and Cys was amplified using polymerase chain reaction (PCR) under the following conditions: initial denaturation at 95 °C for 2 min, followed by a second denaturation at 95 °C for 35 sec, annealing at 52 °C for 35 sec, followed by a cycle extension at 72 °C for 35 sec, for 33 cycles using the light strand primer L4437b (5’-AAGCAGTTGGGCCCATACC-3’; Macey et al. 1997) and heavy strand primer H5934 (5’ AGRGTGCCAATGTCTTTGTGRTT-3’; Macey et al. 1997). PCR products were purified using the AccuPrep PCR Purification Kit (Bioneer, Daejeon, Korea), and were sequenced using the amplifying primers on an ABI 3730 automatic sequencer (Applied Biosystems, CA, USA). Sequences were edited and aligned using Geneious R11 (Biomatters, Ltd, Auckland, New Zealand). All new sequences were deposited in GenBank under accession numbers MT112890–MT112894 (Table 1).

**Table 1.** Specimens used in this study, including locality, collection numbers and Genbank accession numbers. Voucher abbreviations are as follows: Monte L. Bean Life Science Museum at Brigham Young University (BYU), California Academy of Sciences (CAS), the Field Museum of Natural History, Chicago, Illinois, USA (FMNH), La Sierra University Herpetological Collection (LSUHC), Universiti Sains Malaysia Herpetological Collection at the Universiti Sains Malaysia, Penang, Malaysia (USMHC), and Zoological Museum of Kasetsart University (ZMKU).

Species	Locality	Collection number	GenBank accession number	Reference
*Cyrtodactylus bokorensis*	Cambodia, Kampot	FMNH 263228	KT13107	Grismer et al. 2015a
*Hemidactylus garnotii*	Myanmar, Mon State,	CAS 222276	EU68364	Bauer et al. 2008
	Kyait Hti Yo Wildlife Sanctuary			
*Cnemaspis adangrawi*	Thailand, Satun Province,	ZMKU R 00767	MK862112	Ampai et al. 2019
	Mueang Satun District,	THNHM 28207	MK862113	Ampai et al. 2019
	Adang Island	ZMKU R 00770	MK862114	Ampai et al. 2019
	Thailand, Satun Province,	ZMKU R 00775	MK862115	Ampai et al. 2019
	Mueang Satun District, Rawi Island	ZMKU R 00776	MK862116	Ampai et al. 2019
*Cnemaspis affnis*	Malaysia, Penang, Pulau Pinang	LSUHC 6787	KM024682	Grismer et al. 2014
*Cnemaspis argus*	Malaysia, Terengganu,	LSUHC 8304	KM024687	Grismer et al. 2014
	Gunung Lawit	LSUHC 10834	KM024688	Grismer et al. 2014
*Cnemaspis aurantiacopes*	Vietnam, Kien Giang Province, Hon Dat Hill	LSUHC 8610	KM024692	Grismer et al. 2014
		LSUHC 8611	KM024693	Grismer et al. 2014
*Cnemaspis biocellata*	Malaysia, Perlis, Kuala Perlis	LSUHC 8817	KM024707	Grismer et al. 2014
		LSUHC 8817	KM024708	Grismer et al. 2014
	Malaysia, Perlis, Gua Kelam	LSUHC 8789	KM024709	Grismer et al. 2014
*Cnemaspis boulengerii*	Vietnam, Ca Mau Province,	LSUHC 9278	KM024710	Grismer et al. 2014
	Con Dao Archipelago	LSUHC 9279	KM024711	Grismer et al. 2014
*Cnemaspis caudanivea*	Vietnam, Kien Giang Province, Hon Tre Island	LSUHC 8582	KM024714	Grismer et al. 2014
*Cnemaspis chanardi*	Thailand, Nakhon Si Thammarat Province, Thum Thong Panra	LSUHC 9567	KM024715	Grismer et al. 2014
*Cnemaspis chanthaburiensis*	Cambodia, Pursat Province, Phnom Dalai	LSUHC 9338	KM024716	Grismer et al. 2014
*Cnemaspis grismeri*	Malaysia, Perak, Lenggong	LSUHC 9969	KM024722	Grismer et al. 2014
*Cnemaspis hangus*	Malaysia, Pahang, Bukit Hangus	LSUHC 9358b	KM024728	Grismer et al. 2014
*Cnemaspis harimau*	Malaysia, Kedah, Gunung Jeri	LSUHC 9665	KM024730	Grismer et al. 2014
*Cnemaspis huaseesom*	Thailand, Kanchanaburi Province, Sai Yok National Park	LSUHC 9455	KM024733	Grismer et al. 2014
		LSUHC 9457	KM024734	Grismer et al. 2014
		LSUHC 9458	KM024735	Grismer et al. 2014
*Cnemaspis karsticola*	Malaysia, Kelantan, Gunung Reng	LSUHC 9054	KM024736	Grismer et al. 2014
		LSUHC 9055	KM024737	Grismer et al. 2014
*Cnemaspis kumpoli*	Malaysia, Perlis, Perlis State Park	LSUHC 8847	KM024745	Grismer et al. 2014
		LSUHC 8848	KM024746	Grismer et al. 2014
*Cnemaspis lineatubercularis* sp. nov.	Thailand, Nakhon Si Thammarat Province, Lan Saka District, Wang Mai Pak Waterfall	ZMKU R 00825	MT112890	This study
		ZMKU R 00828	MT112891	This study
		ZMKU R 00829	MT112892	This study
		ZMKU R 00830	MT112893	This study
		ZMKU R 00832	MT112894	This study
*Cnemaspis lineogularis*	Thailand, Prachuap Khiri Khan Province,	BYU 62535	KY091231	Wood et al. 2017
	Kui Buri District, Wat Khao Daeng	ZMKU R 00728	KY091233	Wood et al. 2017
*Cnemaspis mahsuriae*	Malaysia, Kedah, Pulau Langkawi, Gunung Raya	LSUHC 11829	KT250634	Grismer et al. 2015b
*Cnemaspis mcguirei*	Malaysia, Perak, Bukit Larut	LSUHC 8853	KM024751	Grismer et al. 2014
*Cnemaspis monachorum*	Malaysia, Kedah, Langkawi Archipelago, Pulau Langkawi	LSUHC 9114	KM024754	Grismer et al. 2014
		LSUHC 10807	KM024755	Grismer et al. 2014
*Cnemaspis narathiwatensis*	Malaysia, Perak, Belum-Temengor, Sungai Enam	USMHC 1347	KM024762	Grismer et al. 2014
		USMHC 1348	KM024763	Grismer et al. 2014
*Cnemaspis neangthyi*	Cambodia, Pursat Province, O’Lakmeas	LSUHC 8515	KM024767	Grismer et al. 2014
		LSUHC 8516	KM024768	Grismer et al. 2014
*Cnemaspis niyomwanae*	Thailand, Trang Province,	LSUHC 9568	KM024773	Grismer et al. 2014
	Thum Khao Ting	LSUHC 9571	KM024774	Grismer et al. 2014
*Cnemaspis nuicamensis*	Vietnam, An Giang Province, Nui Cam Hill	LSUHC 8646	KM024775	Grismer et al. 2014
		LSUHC 8647	KM024776	Grismer et al. 2014
		LSUHC 8648	KM024777	Grismer et al. 2014
*Cnemaspis omari*	Thailand, Satun Province, Phuphaphet Cave	LSUHC 9565	KM024780	Grismer et al. 2014
	Malaysia, Perlis, Perlis State Park	LSUHC 9978	KM024779	Grismer et al. 2014
*Cnemaspis perhentianensis*	Malaysia, Terengganu, Pulau Perhentian Besar	LSUHC 8699	KM024820	Grismer et al. 2014
*Cnemaspis phangngaensis*	Thailand, Phangnga Province,	BYU 62537	KY091234	Wood et al. 2017
	Mueang Phangnga District, Khao Chang, Phung Chang Cave	BYU 62538	KY091235	Wood et al. 2017
*Cnemaspis punctatonuchalis*	Thailand, Prachaup Khiri Khan Province, Thap Sakae	BYU 62539	KY091236	Wood et al. 2017
		BYU 62540	KY091237	Wood et al. 2017
*Cnemaspis roticanai*	Malaysia, Kedah, Pulau Langkawi, Gunung Raya	LSUHC 9430	KM024829	Grismer et al. 2014
		LSUHC 9431	KM024830	Grismer et al. 2014
		LSUHC 9439	KM024831	Grismer et al. 2014
*Cnemaspis siamensis*	Thailand, Chumpon Province, Pathio District	LSUHC 9474	KM024838	Grismer et al. 2014
		LSUHC 9485	KM024839	Grismer et al. 2014
*Cnemaspis tarutaoensis*	Thailand, Satun Province,	ZMKU R 00761	MK862117	Ampai et al. 2019
	Mueang Satun District,	ZMKUR 00763	MK862118	Ampai et al. 2019
	Tarutao Island	ZMKU R 00764	MK862119	Ampai et al. 2019
*Cnemaspis thachanaensis*	Thailand, Surat Thani Province,	BYU 62542	KY091239	Wood et al. 2017
	Tha Chana District,	BYU 62543	KY091243	Wood et al. 2017
	Tham Khao Sonk Hill	BYU 62544	KY091244	Wood et al. 2017
*Cnemaspis tucdupensis*	Vietnam, An Giang Province,	LSUHC 8631	KM024852	Grismer et al. 2014
	Tuc Dup Hill	LSUHC 8632	KM024853	Grismer et al. 2014
*Cnemaspis vandeventeri*	Thailand, Ranong Province, Suk Saran District, Naka	BYU 62541	KY091238	Wood et al. 2017

### Phylogenetic analysis

Homologous sequences of 69 *Cnemaspis*, and the outgroups *Cyrtodactylus bokorensis* and *Hemidactylus garnotii* based on Bauer et al. (2008) and Grismer et al. (2015a), were downloaded from GenBank and aligned to the five newly generated *Cnemaspis* sequences using Geneious R11 (Biomatters, Ltd, Auckland, New Zealand). The aligned dataset was partitioned into four partitions consisting of ND2 codon positions and tRNAs.

Phylogenies were reconstructed with the maximum likelihood (ML) criterion using IQ-TREE 1.6.7 (Nguyen et al. 2015) on the IQ-TREE web server (Trifinopoulos et al. 2016). The best-fit model of substitution for each partition was estimated using IQ-TREE’s ModelFinder function (Kalyaanamoorthy et al. 2017) under the Akaike Information Criterion (AIC). The selected models were TIM+F+I+G4 for first, second and third codon partitions, and HKY+F+G4 for the tRNA partition. Bootstrap analysis was performed using the ultrafast bootstrap approximation (Minh et al. 2013) with 1,000 replicates and 0.95 minimum correlation coefficient.

Phylogenies were also reconstructed with Bayesian Inference (BI) in MrBayes v3.2 on XSEDE on the Cyberinfrastructure for Phylogenetic Research (CIPRES; Miller et al. 2010) computer cluster. The best-fit model of substitution was estimated for each partition with jModelTest 2.1.10 (Posada 2008) under AIC. The selected models were GTR+ I+G4 for each ND2 codon partition, and HKY+ I+G4 for the tRNA partition. Two simultaneous runs, each with three heated and one cold chain, were performed using the default priors for 10,000,000 generations, with trees sampled every 1,000 generations from the Markov Chain Monte Carlo (MCMC). Runs were halted after the average standard deviation split frequency was below 0.01 and convergence was assumed. The first 25% of the trees were discarded as burnin using the sumt command. The convergence of the two simultaneous runs, and stationary state of each parameter, were assessed by examining Trace plots and histograms in Tracer v1.6 (Rambaut et al. 2014). Runs were terminated when the effective sample sizes (ESS) of all parameters ≥ 200.

The most likely tree in the ML analysis, and the 50% majority-rule consensus of the sampled trees from the BI analysis, were visualized using FigTree v1.4.3 (Rambaut 2009). Nodes having bootstrap support (BS) of ≥ 95 and posterior probabilities (PP) of ≥ 0.95 were considered to be well-supported (Huelsenbeck and Ronquist 2001; Wilcox et al. 2002). Uncorrected pairwise genetic distances were calculated using MEGA v7.0.26 (Kumar et al. 2016).

## Results

### Morphological analyses

The ANOVA found statistically significant differences in morphometric characters of the Lan Saka samples and four congeners in the *siamensis* group (*p* < 0.05) for all fifteen variables, as did the Tukey’s HSD pairwise (*p* < 0.05; Table 2).

**Figure 1.**
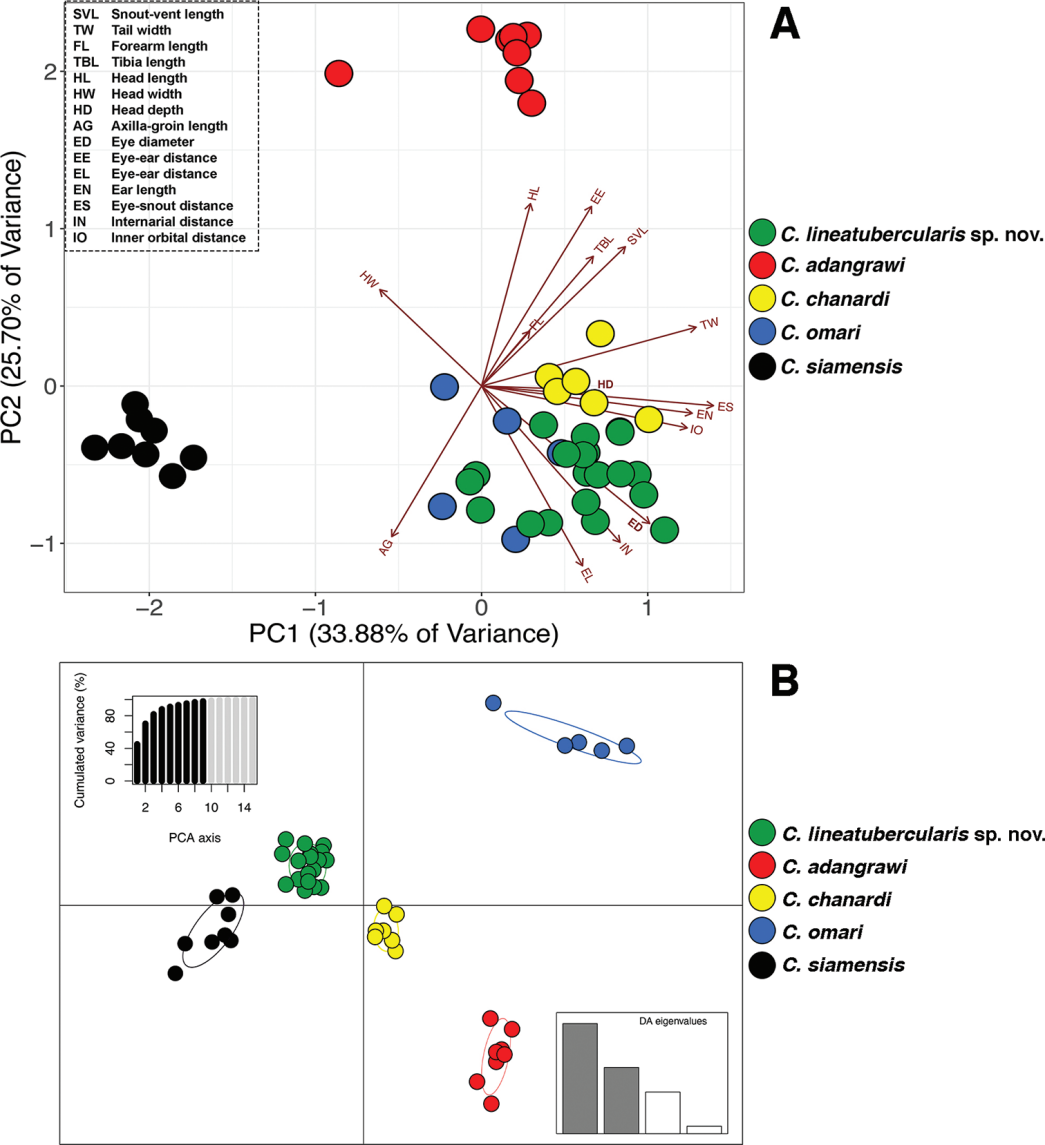
Results of principal component analysis (PCA), and clustering by discriminant function of principal component analysis (DAPC) of 15 morphological variables for 47 individuals of five *Cnemaspis* species (*C. lineatubercularis* sp. nov., *C. adangrawi*, *C. chanardi*, *C. omari*, and *C. siamensis*) **A**PCA scatter plot of PC1 and PC2 showing morphometric differentiation among five species in the *siamensis* group **B**DAPC ordination of all samples showing interspecific variation among five species in the *siamensis* group.

The PCA of five species of *Cnemaspis* showed large morphometric differences on a scatter plot of the first four components with eigenvalues greater than 1.0 (Fig. 1A). These four components accounted for 85.40% of the total variance (Table 3). The first principal component (PC1) accounted for 33.88% of the most of variance and loaded heavily on the head proportions (interorbital distance, eye-nostril distance and eye-snout distance) and the shape of tail (tail width). The second principal component (PC2) accounted for 25.70% and mostly loaded for the body proportion (axillar-groin length) and the head proportions (internarial distance, head length, eye-ear distance and ear length). The third principal component (PC3) accounted for 17.10% and loaded heavily on the head proportions (head width and head depth) and forearm length whereas the fourth (PC4) accounted for 8.72% and loaded heavily on the head proportions (head width, ear length and head length) and the body proportions (axilla-groin length and tibia length). Factor loadings for each component are provided in Table 3. The ordination of the first two components showed separation between the Lan Saka samples and four congeners in the *siamensis* group. The PC2 axis showed separation between *C. adangrawi*, *C. omari*, and *C. siamensis* from *C. chanardi* and the Lan Saka samples. The biplot analysis showed that the Lan Saka samples overlapped slightly with *C. chanardi*. The DAPC (97.70% of cumulative variance) discriminated among groups and supported distinct clusters that corresponded to five *Cnemaspis* species (Fig. 1B).

**Table 2.** Pairwise matrix of significant differences (Tukey’s pairwise; *p <* 0.05) from 15 size-corrected morphometric measurements of *Cnemaspis lineatubercularis* sp. nov. and four congeners of the *siamensis* group including *C. adangrawi*, *C. chanardi*, *C. omari*, and *C. siamensis*. Measurement abbreviations are defined in the text.

Species	*C. lineatubercularis* sp. nov.	*C. adangrawi*	*C. chanardi*	*C. omari*
*C. lineatubercularis* sp. nov.	–	–	–	–
*C. adangrawi*	SVL, FL, TBL, AG, HL, HW, EE, EL, ES, IN, IO	–	–	–
*C. chanardi*	TBL, TW, AG, HL, HW, EL	SVL, FL, TBL, AG, ED, EE, EL, EN, ES, IN, IO	–	–
*C. omari*	FL, TBL, HW, HD, EE, ES, IO	SVL, TW, FL, AG, HL, HW, EE, ED, EL, IN	FL, TBL, HL, HW, HD, ED, EE, ES, IN, IO	–
*C. siamensis*	SVL, TW, FL, TBL, AG, HW, ED, EE, EL, EN, ES, IN, IO	SVL, TW, FL, TBL, AG, HL, EE, EL, EN, ES, IO	SVL, TW, FL, TBL, HL, HD, ED, EE, EL, EN, ES, IN, IO	SVL, TW, FL, TBL, AG, HW, HD, ED, EL, EN, ES

**Table 3.** Summary of eigenvalues, percentage of variance, standard deviation, cumulative proportion, and factor loadings from the principal components (PC) of 15 size-corrected morphometric measurements of *Cnemaspis lineatubercularis* sp. nov. and four congeners of the *siamensis* group including *C. adangrawi*, *C. chanardi*, *C. omari*, and *C. siamensis*. Values highlighted in bold represent those with the greatest contribution to the first four principal components (eigenvalue > 1.0). Measurement abbreviations are defined in the text.

	**PC1**	**PC2**	**PC3**	**PC4**	**PC5**	**PC6**	**PC7**	**PC8**	**PC9**	**PC10**	**PC11**	**PC12**	**PC13**	**PC14**	**PC15**
**Eigenvalue**	5.083	3.855	2.564	1.308	0.520	0.385	0.347	0.237	0.204	0.173	0.131	0.084	0.050	0.037	0.021
% **of Variance**	33.884	25.697	17.097	8.721	3.466	2.565	2.316	1.578	1.362	1.156	0.875	0.558	0.336	0.247	0.142
**Standard deviation**	2.254	1.963	1.601	1.144	0.721	0.620	0.589	0.486	0.452	0.417	0.362	0.289	0.224	0.193	0.146
**Cumulative proportion**	0.339	0.596	0.767	0.854	0.889	0.914	0.937	0.953	0.967	0.978	0.987	0.993	0.996	0.999	1.000
**SVL**	0.254	0.298	0.093	-0.016	0.156	0.841	0.112	-0.264	0.077	-0.008	0.126	-0.034	0.002	0.039	-0.003
**TW**	**0.378**	0.126	-0.028	-0.226	0.142	-0.163	0.093	0.487	0.221	-0.048	0.583	-0.289	-0.078	-0.050	0.119
**FL**	0.085	0.117	**0.532**	0.292	-0.046	-0.103	0.343	0.186	-0.172	-0.080	0.046	-0.006	0.176	0.192	-0.583
**TBL**	0.197	0.277	0.238	**0.498**	0.040	-0.064	0.011	0.160	-0.020	-0.103	-0.325	-0.086	-0.029	-0.473	0.448
**AG**	-0.158	-**0.322**	-0.172	**0.375**	-0.416	0.387	-0.024	0.461	0.140	-0.163	0.039	-0.013	-0.322	0.110	-0.037
**HL**	0.086	**0.390**	-0.181	**0.386**	0.068	-0.232	-0.321	-0.231	0.176	-0.088	0.012	-0.203	-0.234	0.555	-0.033
**HW**	-0.179	0.206	-**0.417**	**0.354**	0.115	-0.051	0.113	-0.072	0.133	0.353	0.289	0.175	-0.008	-0.452	-0.363
**HD**	0.168	-0.007	-**0.516**	0.026	-0.185	-0.063	0.502	-0.196	-0.278	-0.409	-0.078	-0.317	0.155	-0.029	-0.005
**ED**	0.296	-0.294	0.176	0.082	-0.091	-0.161	0.398	-0.359	0.022	0.254	0.112	0.158	-0.586	0.036	0.142
**EE**	0.194	**0.384**	-0.205	-0.088	-0.199	0.009	-0.003	0.278	-0.505	0.148	0.022	0.551	-0.067	0.202	0.145
**ES**	**0.409**	-0.042	-0.010	-0.143	-0.082	-0.050	-0.395	-0.110	0.026	-0.471	-0.055	0.243	-0.239	-0.342	-0.417
**EN**	**0.371**	-0.058	0.030	0.059	-0.639	-0.009	-0.244	-0.144	0.093	0.402	0.064	-0.187	0.394	-0.045	-0.009
**IO**	**0.363**	-0.090	-0.240	-0.057	0.216	-0.003	0.211	0.244	0.459	0.191	-0.553	0.174	0.126	0.170	-0.141
**EL**	0.178	-**0.384**	-0.040	**0.369**	0.248	-0.020	-0.072	-0.104	0.028	-0.239	0.335	0.407	0.441	0.143	0.232
**IN**	0.244	-**0.334**	-0.119	0.149	0.395	0.111	-0.263	0.121	-0.542	0.302	-0.081	-0.341	-0.102	-0.005	-0.150

### Molecular analyses

**Figure 2.**
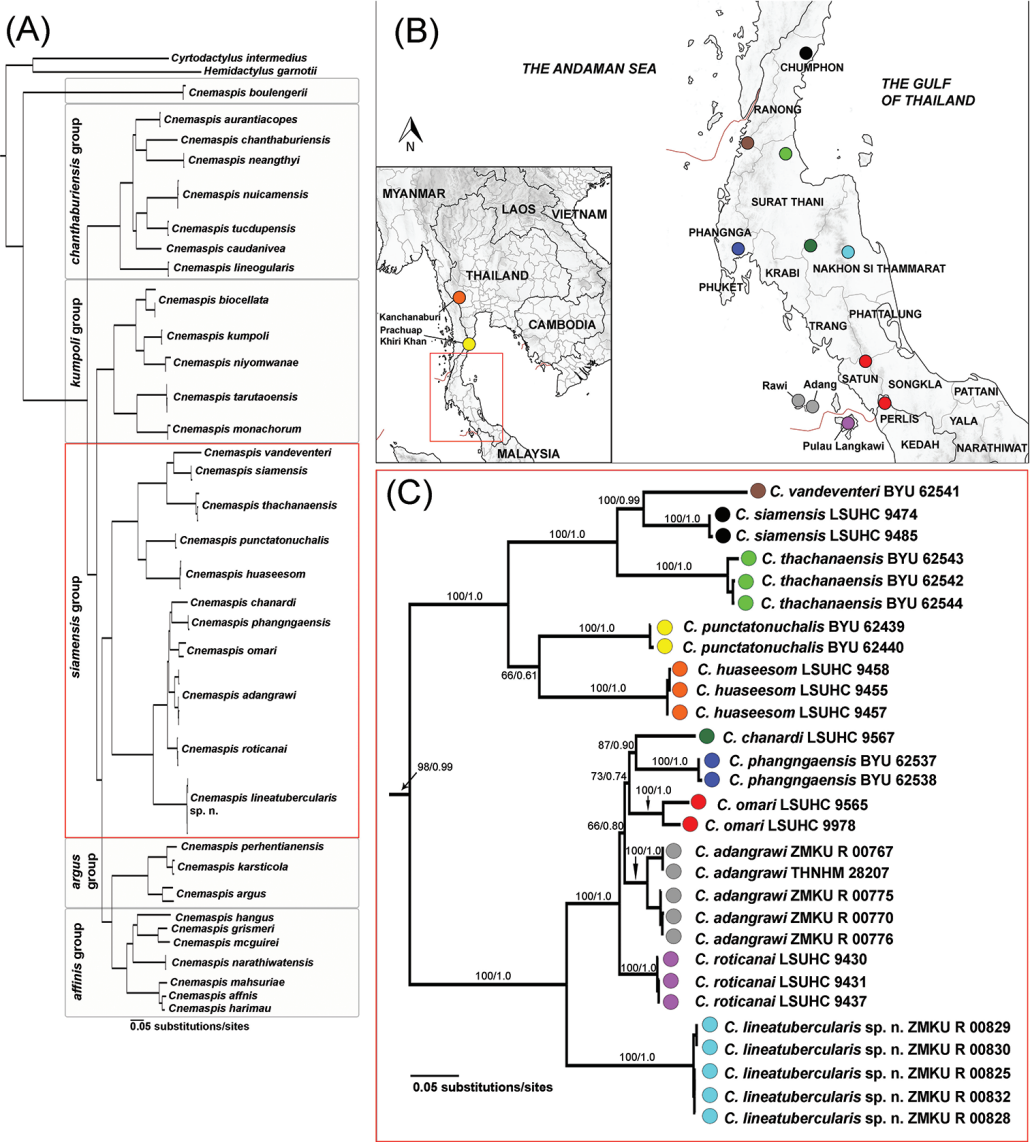
**A** The single best maximum likelihood tree of the mitochondrial NADH dehydrogenase subunit 2 (ND2) gene and flanking tRNAs from geckos of the genera *Cnemaspis*, *Cyrtodactylus* and *Hemidactylus*, shown in full view **B** map illustrating the localities of *Cnemaspis siamensis* group samples used in this study and **C** close-up view of the *C. siamensis* group. Support values at nodes are bootstrap values from a Maximum Likelihood analysis of the same dataset followed by posterior probabilities of the Bayesian Inference analysis.

The aligned dataset contained 1,251 characters of 69 individuals of *Cnemaspis* and two individuals of the outgroup species. The standard deviation of split frequencies among the two simultaneous BI runs was 0.001646. The ESS values were greater than or equal to 2,944 for all parameters. The maximum likelihood value of the best ML tree was lnL = -54,716.041. The most likely ML tree and the 50% majority rule consensus tree from the BI analysis resulted in trees with similar topologies (Fig. 2).

The Lan Saka samples represented a well-supported clade (100 BS, 1.0 PP) within the *siamensis* group and the sister taxon of a clade containing *C. adangrawi*, *C. chanardi*, *C. phangngaensis*, *C. omari*, and *C. roticanai* (Fig. 2), although relationships within that sister clade were not resolved (Fig. 2). Sequence divergences (uncorrected *p*-distance for ND2) ranged from 0.00–0.40% within the Lan Saka samples and 15.53–28.09% among the Lan Saka samples and other species in the *siamensis* group (Table 4).

**Table 4.** Mean (min-max) uncorrected *p*-distances (%) within the *siamensis* group based on 1,251 bp of ND2 and flanking tRNA. Numbers in bold are within species divergence. *N* = number of individuals.

No.	Species	N	1	2	3	4	5	6	7	8	9	10	11
1	*C. lineatubercularis* sp. nov.	5	**0.21**										
			**(0.00–0.40)**										
2	*C. adangrawi*	5	17.78	**2.81**									
			(17.02–18.72)	**(0.00–4.68)**									
3	*C. chanardi*	1	16.98	11.40	**0.00**								
			(16.81–17.23)	(10.85–11.91)									
4	*C. huaseesom*	3	27.45	9.36	11.81	**2.13**							
			(27.23–27.87)	(8.30–10.21)	(11.49–12.13)	**(0.00–4.26)**							
5	*C. omari*	2	18.79	10.19	11.38	11.17	**0.11**						
			(18.51–19.15)	(9.57–10.85)	(11.27–11.49)	(10.85–11.49)	**(0.00–0.21)**						
6	*C. phangngaensis*	2	17.64	25.83	24.40	27.77	25.00	**0.00**					
			(17.45–17.87)	(25.74–25.96)	(24.26–24.68)	(27.66–27.87)	(24.89–25.11)						
7	*C. punctatonuchalis*	2	25.91	8.92	11.77	9.01	8.90	28.16	**0.11**				
			(25.74–26.17)	(8.51–9.57)	(11.70–11.91)	(8.72–9.36)	(8.72–9.15)	(28.09–28.30)	**(0.00–0.21)**				
8	*C. roticanai*	3	15.77	24.26	24.04	26.60	25.21	12.34	26.88	**0.00**			
			(15.53–16.17)	(24.04–24.47)	(24.04–24.04)	(25.96–27.23)	(25.11–25.32)	(12.34–12.34)	(26.81–27.02)				
9	*C. siamensis*	2	27.74	25.50	24.40	28.30	26.13	13.35	27.66	14.47	**0.53**		
			(27.66–27.87)	(25.10–25.96)	(24.26–24.68)	(27.23–28.94)	(25.74–26.81)	(13.19–14.26)	(27.45–28.09)	(14.26–14.89)	**(0.00–1.06)**		
10	*C. thachanaensis*	3	27.53	25.23	25.53	26.38	25.00	19.36	25.60	21.06	21.13	**0.00**	
			(27.23–28.09)	(24.04–26.17)	(25.53–25.53)	(26.38–26.38)	(25.00–25.00)	(19.36–19.36)	(25.53–25.74)	(21.06–21.06)	(21.06–21.28)		
11	*C. vandeventeri*	1	25.62	26.00	26.17	28.19	23.72	19.36	27.52	20.64	20.99	16.95	**0.43**
			(25.53–25.74)	(25.74–26.38)	(26.17–26.17)	(27.87–28.51)	(23.62–23.83)	(19.36–19.36)	(27.45–27.66)	(20.64–20.64)	(20.64–21.70)	(16.81–17.02)	**(0.00–0.64)**

### Taxonomic hypotheses

*Cnemaspis* samples from Lan Saka District, Nakhon Si Thammarat Province, are diagnosable in the morphological and molecular analyses. Based on these corroborated, independent lines of evidence, we hypothesize that the Lan Saka samples represent a new species that is described as follows.

## Systematics

### Cnemaspis lineatubercularis sp. nov.

Taxon classificationAnimaliaSquamataGekkonidae

F5F65232-3BE6-5D38-988B-AA26777C79EE

http://zoobank.org/B789936F-0A24-4977-B200-4CF1D67B20FF

**Figure 3.**
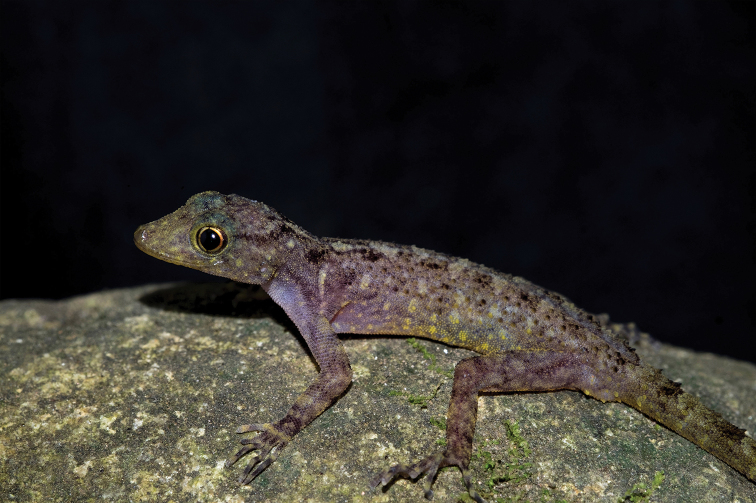
Male holotype (ZMKU R 00828) of *Cnemaspis lineatubercularis* sp. nov. from Wang Mai Pak Waterfall, Lan Saka District, Nakhon Si Thammarat Province, Thailand.

**Figure 4.**
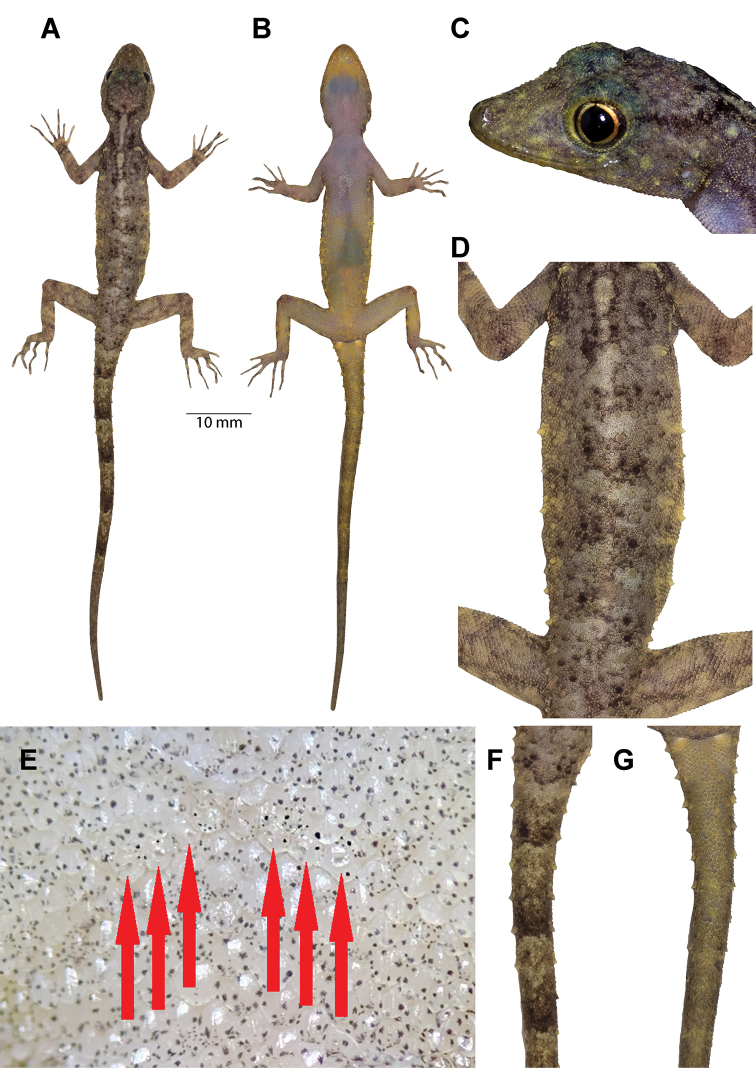
Male holotype (ZMKU R 00828) of *Cnemaspis lineatubercularis* sp. nov. from Wang Mai Pak Waterfall, Lan Saka District, Nakhon Si Thammarat Province, Thailand, in life **A** dorsal view **B** ventral view **C** lateral view of the head **D** dorsal view of trunk **E** precloacal region showing distribution of pore-bearing scales (red arrows) **F** dorsal view of tail **G** ventral view of tail. Scale bar: 10 mm (in dorsal and ventral views).

**Figure 5.**
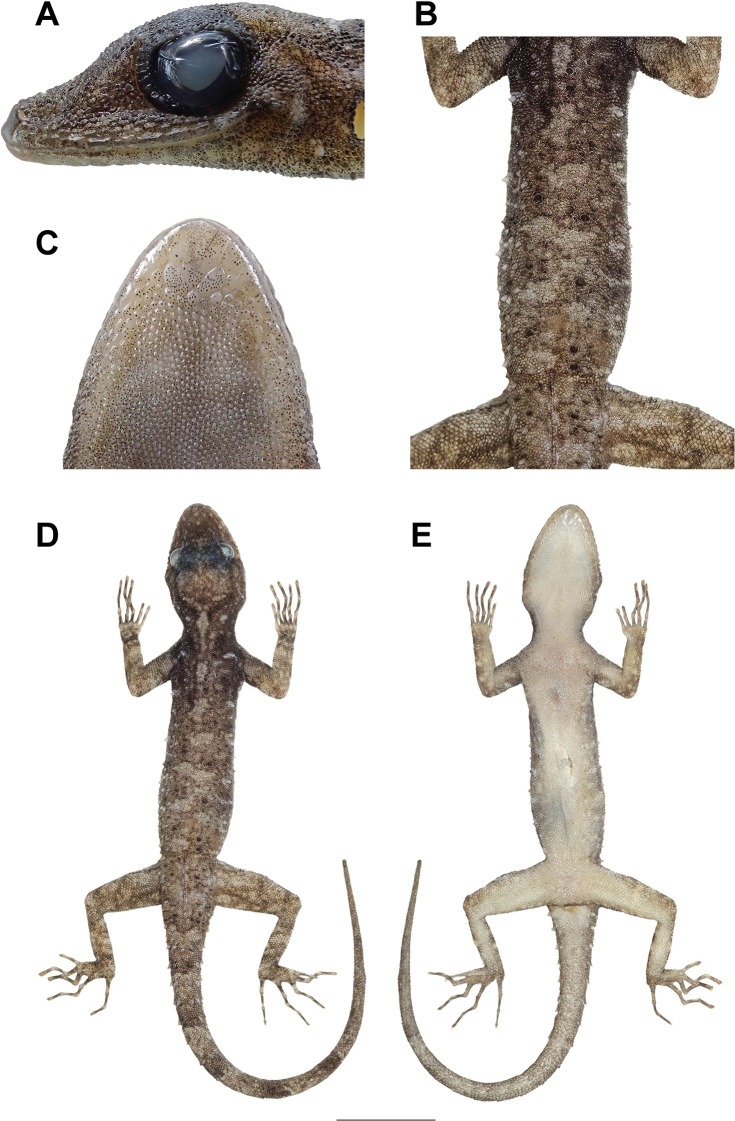
Male holotype (ZMKU R 00828) of *Cnemaspis lineatubercularis* sp. nov. from Wang Mai Pak Waterfall, Lan Saka District, Nakhon Si Thammarat Province, Thailand, in preservative **A** lateral view of head **B** dorsal view of trunk **C** ventral view of chin **D** dorsal view **E** ventral view. Scale bar: 10 mm (**D, E**).

**Figure 6.**
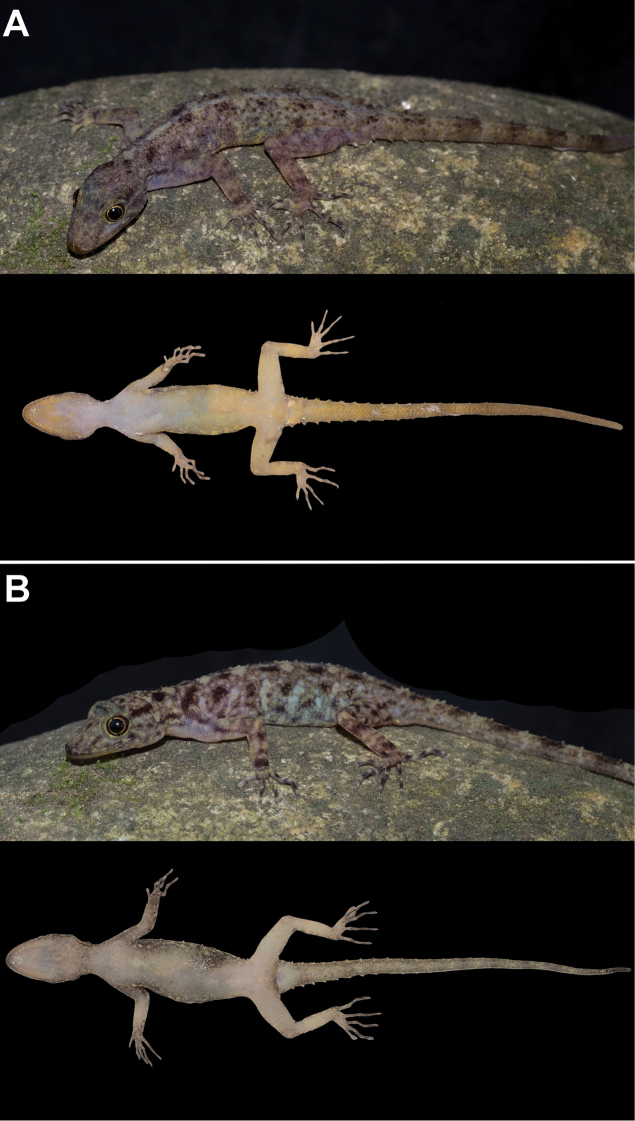
Coloration of *Cnemaspis lineatubercularis* sp. nov. in dorsal (above) and ventral (below) views of **A** male paratype ZMKU R 00830 and **B** female paratype ZMKU R 00835. Note yellowish ventral coloration that is present in males but absent in females.

**Figure 7.**
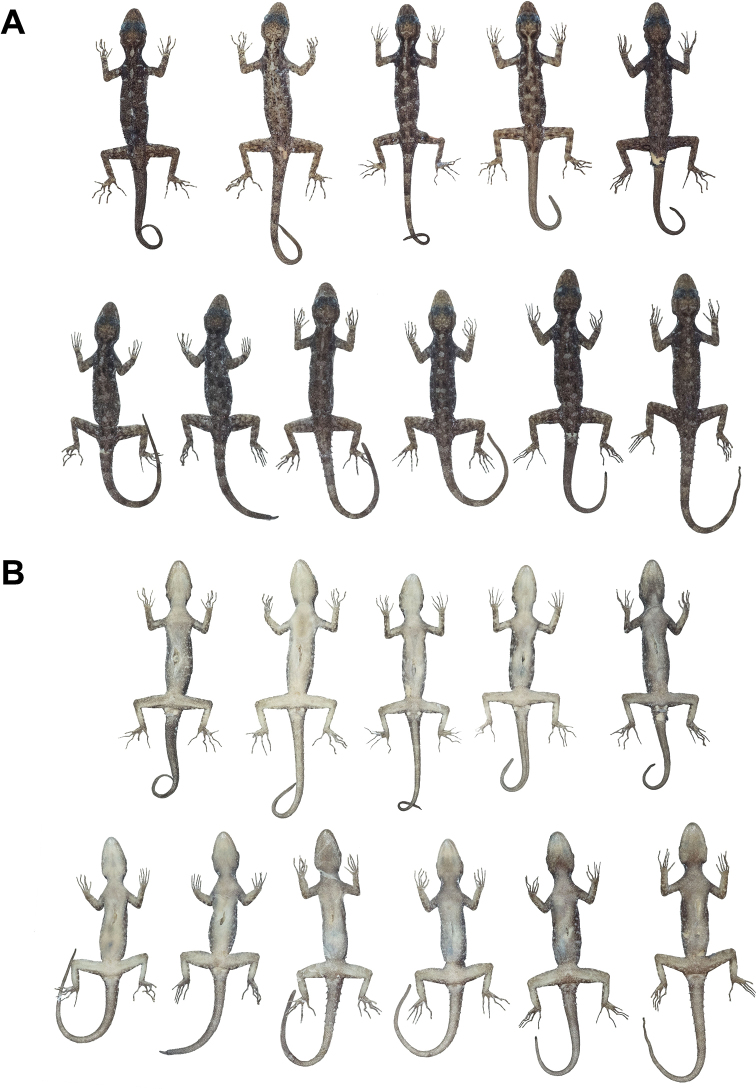
Male paratypes of *Cnemaspis lineatubercularis* sp. nov. in preservative in **A** dorsal view **B** ventral view; from left to right, top panel: ZMKU R 00821, ZMKU R 00822, ZMKU R 00823, ZMKU R 00824 and ZMKU R 00825; bottom panel: ZMKU R 00827, ZMKU R 00829, ZMKU R 00830, ZMKU R 00831, THNHM 28694 and THNHM 28695.

**Figure 8.**
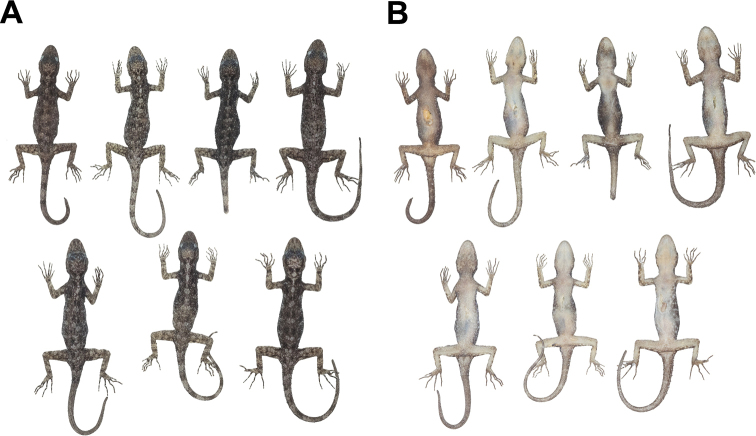
Female paratypes of *Cnemaspis lineatubercularis* sp. nov. in preservative in **A** dorsal view **B** ventral view; from left to right, top panel: ZMKU R 00826, ZMKU R 00832, THNHM 28696 and THNHM 28697; bottom panel: ZMKU R 00833, ZMKU R 00834 and ZMKU R 00835.

Figures 3 , 4 , 5 , 6 , 7 , 8

#### Type material.

***Holotype*** (Figs 3–5). ZMKU R 00828, adult male from Thailand, Nakhon Si Thammarat Province, Lan Saka District, Kam Lon Subdistrict, Wang Mai Pak Waterfall (8°26.807'N, 99°46.525'E; 96 m a.s.l.), collected on 25 January 2019 by Natee Ampai, Anchalee Aowphol, Attapol Rujirawan, Korkwan Termprayoon and Siriporn Yodthong.

***Paratypes*** (Figs 6–8). Eighteen paratypes (adult males = 11, adult females = 7). ZMKU R 00821–00825 (five adult males), and ZMKU R 00826 (adult female), same data as holotype except that they were collected on 25 October 2016. ZMKU R 00827, ZMKU R 00829–00831 (four adult males), ZMKU R 00832–00835 (four adult females), THNHM 28694–28695 (two adult males) and THNHM 28696–28697 (two adult females), same data as holotype.

#### Diagnosis.

*Cnemaspis lineatubercularis* sp. nov. can be distinguished from all other *Cnemaspis* by having the following combination of characters: (1) maximum SVL of 40.6 mm (mean 38.8 ± SD 1.4, *N* = 12) in adult males and maximum SVL of 41.8 mm (mean 39.5 ± SD 1.9, *N* = 7) in adult females; (2) 8–9 supralabial and infralabial scales; (3) gular, pectoral, abdominal, and subcaudal scales keeled; (4) rostral, interorbitals, supercilium, palmar scales, and ventral scales of brachia smooth; (5) 5–6 small, subconical spine-like tubercles present on flanks (6) 19–21 paravertebral tubercles linearly arranged; (7) 27–29 subdigital lamellae under the 4^th^ toe; (8) 4–7 pore-bearing precloacal scales, pores rounded, arranged in chevron shape and separated in males; (9) one postcloacal tubercle each side in males; (10) ventrolateral caudal tubercles anteriorly present; (11) caudal tubercles restricted to a single paravertebral row on each side; (12) single median row of subcaudal scales keeled and lacking enlarged median row; and (13) gular region, abdomen, limbs and subcaudal region yellowish only in males. These differences are summarized among geographically close congeners in the *siamensis* group (Table 5).

**Table 5.** Meristic character state and color pattern of species in the *Cnemaspis siamensis* group. Measurements are taken in millimeters and measurement abbreviations are defined in the text. Key: – = data unavailable, w = weak.

Characters/Species	*C. lineatubercularis* sp. nov.	*C. adangrawi*	*C. chanardi*	*C. huaseesom*	*C. kamolnorranathi*	*C. omari*	*C. phangngaensis*	*C. punctatonuchalis*	*C. roticanai*	*C. siamensis*	*C. thachanaensis*	*C. vandeventeri*
Sample size	19	15	25	5	3	8	2	5	8	12	6	3
Maximum SVL	41.8	44.9	40.9	43.5	37.8	41.3	42.0	49.6	47.0	39.7	39.0	44.7
Supralabial scales	8–9	10	8–10	7–10	8–9	8–9	10	8	8–9	8–9	10–11	8–9
Infralabial scales	8–9	9	8	6–9	7–8	7–8	10	7–8	7–8	6–8	9–11	7–9
Ventral scales keeled (1) or smooth (0)	1	1	1	0	w,0	1	1	0	1	1	1	1
No. of pore-bearing precloacal scales	4–7	6–8	6–8	5–8	6–7	3–6	4	0	3–6	0	0	4
Precloacal scales pore-bearing continuous (1) or separated (0)	0	0	0	1	1	0	1	–	0	–	–	0
Precloacal pores elongate (1) or round (0)	0	0	0	0	1	0	0	–	0	–	–	0
No. of paravertebral tubercles	19–21	23–25	22–25	18–24	19–24	22–29	22	24–27	25–27	19–25	15–19	25–29
Paravertebral tubercles linearly arranged (1) or more random (0)	1	0	0	w,0	w	w,0	1	w	0	0	1	0
Tubercles present (1) or absent (0) on lower flanks	1	0	1	1	1	w,1	0	1	1	1	1	1
No. of 4^th^ toe lamellae	27–29	26–28	26–29	21–31	24–28	25–28	29	29–31	26–29	24–26	24	24–28
Ventrolateral caudal tubercles anteriorly present (1) or not (0)	1	1	0	0	0	0	1	1	0	0	1	0
Lateral caudal furrows present (1) or absent (0)	1	1	1	1	1	1	1	1	1	1	1	0
Subcaudal keeled (1) or smooth (0)	1	1	1	0	1	1	1	0	1	1	1	1
Single median row of keeled subcaudals (1) or smooth (0)	1	1	0	0	w	0	1	0	0	0	1	w
Enlarge median subcaudal scales row (1) or not (0)	0	0	1	0	w	0	0	1	w	1	0	1
Caudal tubercles restricted to a single paravertebral row on each side (1) or not (0)	1	0	0	0	0	0	1	0	0	0	1	0
No. of postcloacal tubercles in males	1	1	1	1–2	1–2	1	2	1–3	1–2	1–2	0	1–3
Subtibial scales keeled (1) or smooth (0)	1	1	1	0	0,1	1	1	1	1	1	1	1
Subcaudal region yellow present (1) or not (0)	1	0	1	1	0	1	1	0	1	0	0	0
Ventral pattern sexually dimorphic present (1) or not (0)	1	1	1	1	–	1	1	1	1	1	1	1
Dorsal color pattern sexually dimorphic (1) or not (0)	0	0	0	1	0	0	1	1	1	0	0	0

#### Description of holotype.

Adult male; SVL 40.1 mm; head moderate in size (HL/SVL 0.26), narrow (HW/SVL 0.16), flattened (HD/HL 0.41) and head distinct from neck; snout moderate (ES/HL 0.47), snout slightly concave in lateral view; postnasal region concave medially; scales of rostrum smooth, larger than conical scales on occiput; weak supraorbital ridges; gular marking absent; gular and throat scales granular, keeled and round; shallow frontorostral sulcus; canthus rostralis nearly absent, smoothly rounded; eye large (ED/HL 0.23); pupil round; extral-brillar fringe scales largest anteriorly; scales on interorbitals and supercilium smooth; ear opening oval, taller than wide; rostral slightly concave; rostral bordered posteriorly by supranasals and laterally by first supralabials; 9, 9 (Right, Left) supralabials decreasing in size posteriorly; 9, 9 (Right, Left) infralabials decreasing in size posteriorly; nostril elliptical, oriented dorsoposteriorly, bordered posteriorly by small postnasal scales; mental scales large, triangular, concave, bordered posteriorly by three large postmentals.

Body slender, elongate (AG/SVL 0.43); small, keeled, dorsal scales equal in size throughout body intermixed with several large, keeled, multicarinate tubercles; 19 paravertebral tubercles linearly arranged; tubercles present on lower flanks; tubercles extend from occiput to tail; five small, subconical spine-like tubercles on flanks; dorsal scales raised and keeled; pectoral and abdominal scales keeled, round, flat to concave, slightly larger than dorsal and not larger posteriorly; ventral scales of brachia smooth, raised and juxtaposed; six separated pore-bearing precloacal scales with rounded pores; precloacal depression absent; femoral pores absent.

Fore and hind limbs moderately long, slender; scales beneath forearm slightly raised, smooth and sub-imbricate; subtibial scales keeled; palmar scales smooth and juxtaposed; digits elongate, slender, inflected joint and bearing slightly recurved claws; subdigital lamellae unnotched; lamellae beneath first phalanges wide; lamellae beneath phalanx immediately following inflection granular; lamellae of distal phalanges wide; lamellae beneath inflection large; interdigital webbing absent; enlarged submetatarsal scales on 1^st^ toe absent; total subdigital lamellae on fingers: 17-21-25-28-26 (right manus), 17–16 (broken)-25-28-26 (left manus); fingers increase in length from first to fourth with fourth and fifth nearly equal in length; relative length of fingers IV>V>III>II>I; total subdigital lamellae on toes: 13-21-24-29-25 (right pes), 13-21-24-29-25 (left pes); toes increase in length from first to fifth with fourth and fifth nearly equal in length; relative length of toes IV>V>III>II>I.

The original tail cylindrical, swollen at the base and longer than head and body (TL/SVL 1.36); subcaudal scales keeled, juxtaposed, similar to dorsal scale of the tail size; shallow, middorsal furrow; deeper lateral caudal furrow present; enlarged, transverse caudal tubercles arranged in segmented whorls, not encircling tail; enlarged median subcaudal scale row absent; caudal tubercles absent from lateral furrow; tail length (TL) 54.7 mm; a single postcloacal tubercle on each side at lateral surface of hemipenial swellings at the base of tail.

#### Measurements of holotype

(in mm; Table 6). SVL 40.1; TL (original) 54.7; TW 3.9; FL 5.8; TBL 7.2; AG 17.4; HL 10.3; HW 6.3; HD 4.2; ED 2.4; EE 3.1; ES 4.8; EN 3.9; IO 2.9; EL 1.0; IN 1.0.

**Table 6.** Descriptive measurements in millimeters and characters of the type series of *Cnemaspis lineatubercularis* sp. nov. Key: H = holotype; P = paratype; M = male; F = female; – = data unavailable or absent; b = broken; r = regenerated. Measurement abbreviations are defined in the text.

Museum number	ZMKU R 00828	ZMKU R 00821	ZMKU R 00822	ZMKU R 00823	ZMKU R 00824	ZMKU R 00825	ZMKU R 00827	ZMKU R 00829	ZMKU R 00830	ZMKU R 00831	THNHM 28694	THNHM 28695	ZMKU R 00826	ZMKU R 00832	THNHM 28696	THNHM 28697	ZMKU R 00833	ZMKU R 00834	ZMKU R 00835
Type series	H	P	P	P	P	P	P	P	P	P	P	P	P	P	P	P	P	P	P
Sex	M	M	M	M	M	M	M	M	M	M	M	M	F	F	F	F	F	F	F
SVL	40.1	39.5	40.1	37.8	37.6	39.1	37.7	36.7	37.0	39.4	40.6	39.9	37.3	38.3	38.8	41.8	41.4	37.5	41.1
TL	54.7	39.7r	47.3r	37.4r	38.9r	39.2r	49.1	32.6r	33.9r	45.9r	39.5r	55.4	37.3r	47.6r	17.6b	56.1	44.35r	53.3	51.5
TW	3.9	4.0	4.1	3.8	3.7	4.0	3.6	3.7	3.8	3.9	4.1	3.9	3.6	3.8	3.6	4.2	4.0	3.8	3.9
FL	5.8	5.8	5.8	5.6	5.6	5.7	5.7	5.5	5.6	5.7	5.8	5.8	5.6	5.7	5.6	5.8	5.7	5.6	5.8
TBL	7.2	7.2	7.3	7.2	7.1	7.2	7.2	7.1	7.1	7.2	7.3	7.2	7.1	7.1	7.2	7.3	7.3	7.1	7.2
AG	17.4	17.5	17.6	17.3	17.3	17.5	17.3	16.8	16.9	17.4	17.6	17.5	17.4	17.5	17.4	17.6	17.5	17.3	17.5
HL	10.3	10.2	10.3	10.0	10.1	10.2	10.1	10.1	10.2	10.3	10.4	10.4	9.9	10.2	10.1	10.4	10.4	10.0	10.4
HW	6.3	6.2	6.3	6.1	6.1	6.2	6.1	6.0	6.0	6.2	6.3	6.3	6.1	6.2	6.2	6.3	6.2	6.1	6.3
HD	4.2	4.2	4.2	4.0	3.8	4.2	3.8	3.9	4.0	4.1	4.2	4.1	3.8	4.0	3.9	4.3	4.2	4.0	4.2
ED	2.4	2.3	2.4	2.2	2.2	2.3	2.1	2.1	2.2	2.3	2.4	2.3	2.1	2.2	2.3	2.4	2.4	2.3	2.4
EE	3.1	3.0	3.1	2.8	2.8	3.0	2.9	2.8	2.9	3.1	3.1	3.1	2.9	3.0	2.9	3.2	3.2	3.1	3.2
ES	4.8	4.9	5.1	4.7	4.7	4.9	4.7	4.7	4.7	5.1	5.1	4.8	4.6	4.8	4.7	5.1	5.1	4.8	5.1
EN	3.9	3.6	3.8	3.4	3.4	3.5	3.4	3.6	3.6	3.8	3.9	3.8	3.4	3.7	3.7	3.9	3.9	3.7	3.8
IO	2.9	2.8	2.9	2.8	2.8	2.9	2.6	2.7	2.8	2.8	2.9	2.8	2.6	2.7	2.6	2.9	2.9	2.7	2.9
EL	1.0	1.1	1.1	1.0	1.0	1.0	1.0	0.9	0.9	1.1	1.1	1.0	1.0	1.0	0.9	1.0	1.0	1.0	1
IN	1.0	1.1	1.0	1.0	1.0	1.0	1.0	1.0	0.9	1.0	1.1	1.0	1.0	1.1	1.0	1.1	1.1	1.1	1.1
SUP	9	9	9	9	9	9	9	9	8	9	9	9	9	9	9	9	9	9	9
INF	9	8	8	8	8	8	8	8	8	9	8	8	9	9	9	9	9	9	9
Pore-bearing precloacal scales	6	4	4	4	5	4	4	4	5	5	7	4	–	–	–	–	–	–	–
PVT	19	20	20	19	20	20	20	19	20	21	20	20	19	21	20	19	20	21	19
Spine-like tubercles on flank	5	5	6	5	6	6	6	6	6	5	6	6	6	6	5	5	6	6	5
4^th^ toe lamellae	29	29	29	28	28	28	27	28	28	27	27	29	29	27	27	28	27	28	27

#### Coloration in life

(Fig. 4). Dorsal ground color of head light brown, top of head bearing small, diffuse, faint black and yellowish markings; dark postorbital stripes faint, extending to nape; large, round, whitish marking on nape; single light-yellowish prescapular crescent on the shoulder, located dorsoanteriorly of forelimb insertion; dorsal ground color of body, limbs and tail light brown with black irregular blotches; ground color of ventral surfaces grayish-white intermixed with yellowish blotches; ventral pattern sexually dimorphic, anterior gular, abdominal, and caudal regions yellowish in males; two dark blotches on nape form a bipartite pattern; light sage vertebral blotches extending from the nape to tail; flanks with irregular, incomplete brown to yellowish blotches becoming smaller posteriorly; tubercles on the whole body white or yellow; widely separated, white or yellow tubercles occur on flanks; subconical spine-like yellowish tubercles on flanks; limbs beige with dark brown mottling; tail faintly marked with dark brown.

#### Coloration in preservative

**(Figs 5, 7, 8).** Color pattern similar to that in life with some fading of markings. Dorsal ground color of head, body, limbs and tail darker brown than in life, with indistinct, irregular markings. Yellow coloration in gular, pectoral, abdominal regions, flanks, and tail faded to light-yellow and creamy-white.

#### Variation.

Most paratypes approximate the holotype in general aspects of morphology (Figs 6–8), with most differences found in the degree of vertebral blotches. All adult female paratypes lack the yellowish coloration in the gular, abdominal, and caudal regions. ZMKU R 00821–00825, ZMKU R 00829–00831, THNHM 28694 (nine adult males) and ZMKU R 00826, ZMKU R 00832 and ZMKU R 00833 (three adult females) have regenerated tails of uniform tan coloration. ZMKU R 00821, 00824, 00827 (three adult males) and ZMKU R 00832, 00835 (two adult females) have lighter dorsal markings that appear more as transverse bands than as paravertebral blotches. THNHM 28696 (adult female) has a broken tail. Differences in meristic and morphometric characters within the type series are presented in Table 6.

#### Distribution and natural history.

**Figure 9.**
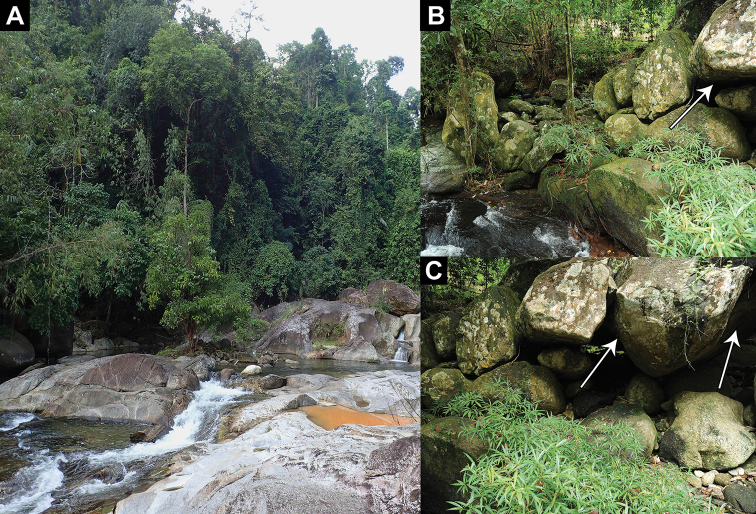
Habitats of *Cnemaspis lineatubercularis* sp. nov **A** Wang Mai Pak Waterfall at type locality **B** microhabitat of holotype in granitic rocky stream (white arrow) **C** microhabitat of paratypes in granitic rocky outcrops (white arrows) at Wang Mai Pak Waterfall, Lan Saka District, Nakhon Si Thammarat Province, Thailand.

*Cnemaspis lineatubercularis* sp. nov. is known only from Wang Mai Pak Waterfall (96 m a.s.l.), Kam Lon Subdistrict, Lan Saka District, Nakhon Si Thammarat Province, southern Thailand (Fig. 9). The type locality is surrounded by lowland evergreen forest along a river basin in the southern part of the Nakhon Si Thammarat mountain range. Specimens were found only along granitic rocky streams of Wang Mai Pak Waterfall. The rocky boulder microhabitats of this species are dry with cool surface temperatures (24.8–26.7 °C, 73.2–86.1% relative humidity). When disturbed, some individuals retreated deeper into rock crevices, cracks, more shaded areas or beneath rock boulders.

Seven specimens (ZMKU R 00822–00825, ZMKU R 00827, THNHM 28696–28697) were collected during the day (1650–1847 h) and 12 specimens (ZMKU R 00821, ZMKU 00826, ZMKU R 00828–00832, THNHM 28694–28695 and ZMKU R 00833–00835) were collected at night (1913–1951 h).

The male holotype was found during the night (1943 h) perched head down on a vertical surface in a crevice of a granitic rock boulder near a stream. A female paratype (ZMKU R 00832) was found with the male holotype, separated by only a distance of approximately 10 cm.

Paratypes that were found during the day were in shaded areas, crevices of boulders, rock walls and on boulder outcrops near streams. Paratypes found at night were in shaded surfaces of the boulders, within deep crevices, or perched on vegetation near a rocky stream. Three gravid females (ZMKU R 00832–00834) contained one or two eggs during January 2019. Some juveniles (SVL < 30 mm; not collected) were found in rock cracks and perched on a rock near a stream on 25 January 2019.

*Cnemaspis lineatubercularis* sp. nov. appears to be a diurnal species in that observed specimens during daytime were active and fast-moving when disturbed, but those at night were inactive, slow-moving or asleep on dry granitic rocks and vegetations. At night, *Cyrtodactylus lekaguli* and *Gehyra mutilata* were found in syntopy with the new species on a rock wall and vegetation near a stream. A summary of ecological parameters of activity periods, elevation (lowland < 600 m), microhabitat preference and presence or absence of ocelli (eyespots) of *Cnemaspis* in Thailand is shown in Table 7.

**Table 7.** Ecological parameters of activity period, elevation (lowland < 600 m), microhabitat preference and presence or absence of ocelli (eyespots) in 18 species of *Cnemaspis* in Thailand based on this and previous studies (Grismer et al. 2010, 2014; Wood et al. 2017; Ampai et al. 2019).

Species/ Parameters	Activity period		Elevation		Microhabitat preference				Ocelli location		
	Diurnal	Nocturnal	Lowland	Upland	Granite	Limestone	Vegetation	Terrestrial	Head	Neck	Shoulders
***affinis* group**											
*C. narathiwatensis*	X		X	X	X						
***chanthaburiensis* group**											
*C. chanthaburiensis*		X	X	X				X			
*C. lineogularis*	X		X			X					
***kumpoli* group**											
*C. biocellata*	X		X			X			X	X	X
*C. kumpoli*		X	X		X						X
*C. niyomwanae*		X	X			X					
*C. tarutaoensis*	X		X			X					
***siamensis* group**											
*C. lineatubercularis* sp. nov.	X		X		X		X				
*C. adangrawi*	X		X		X		X				
*C. chanardi*	X		X		X		X				
*C. huaseesom*		X	X			X					
*C. kamolnorranathi*		X	X		X	X	X	X			
*C. omari*	X		X				X				
*C. phangngaensis*	X		X			X	X				
*C. punctatonuchalis*		X	X		X					X	X
*C. siamensis*	X		X				X				
*C. thachanaensis*	X		X			X	X				
*C. vandeventeri*		X	X		X		X				

#### Etymology.

The specific epithet *lineatubercularis* is taken from *linea* (Lat. for line) and *tubercularis* (Lat. for having tubercles), in reference to the new species having paravertebral tubercles linearly arranged.

#### Comparisons.

*Cnemaspis lineatubercularis* sp. nov. can be distinguished from other members of the *siamensis* group (*C. adangrawi*, *C. chanardi*, *C. huaseesom*, *C. kamolnorranathi*, *C. omari*, *C. phangngaensis*, *C. punctatonuchalis*, *C. roticanai*, *C. siamensis*, *C. thachanaensis*, and *C. vandeventeri*; Table 5) by having a smaller maximum SVL of 41.8 mm (vs. 44.9 mm in *C. adangrawi*, 43.5 mm in *C. huaseesom*, 42.0 mm in *C. phangngaensis*, 49.6 mm in *C. punctatonuchalis*, 47.0 mm in *C. roticanai*, 44.7 mm in *C. vandeventeri*) and by having a larger maximum SVL 41.8 mm (vs. 40.9 mm in *C. chanardi*, 37.8 mm in *C. kamolnorranathi*, 41.3 mm in *C. omari*, 39.7 mm in *C. siamensis*, 39.0 mm in *C. thachanaensis*).

*Cnemaspis lineatubercularis* sp. nov. is distinguished from *C. adangrawi*, *C. phangngaensis*, and *C. thachanaensis* by having fewer 8–9 supralabial scales (vs. 10 in *C. adangrawi* and *C. phangngaensis*, 10–11 in *C. thachanaensis*). This species is distinguished from *C. phangngaensis* by having fewer 8–9 infralabial scales (vs. 10 in *C. phangngaensis*). This species is distinguished from *C. huaseesom* and *C. punctatonuchalis* by having keeled ventral scales (vs. smooth ventral scales in *C. huaseesom* and *C. punctatonuchalis*). This species is distinguished from *C. punctatonuchalis*, *C. siamensis*, and *C. thachanaensis* by presence of precloacal pores (vs. precloacal pores absent in *C. punctatonuchalis*, *C. siamensis*, *C. thachanaensis*). This species is distinguished from *C. huaseesom*, *C. kamolnorranathi*, and *C. phangngaensis* by having a separated row of precloacal pores (vs. continuous in *C. huaseesom*, *C. kamolnorranathi*, *C. phangngaensis*). This species is distinguished from *C. kamolnorranathi* by having rounded precloacal pores (vs. pores elongated in *C. kamolnorranathi*).

*Cnemaspis lineatubercularis* sp. nov. is distinguished from *C. adangrawi*, *C. chanardi*, *C. omari*, *C. phangngaensis*, *C. punctatonuchalis*, *C. roticanai*, and *C. vandeventeri* by having fewer 19–21 paravertebral tubercles (vs. 23–25 in *C. adangrawi*, 22–25 in *C. chanardi*, 22–29 in *C. omari*, 22 in *C. phangngaensis*, 24–27 in *C. punctatonuchalis*, 25–27 in *C. roticanai*, 25–29 in *C. vandeventeri*). This species is distinguished from *C. adangrawi*, *C. chanardi*, *C. huaseesom*, *C. omari*, *C. roticanai*, *C. siamensis*, and *C. vandeventeri* by having paravertebral tubercles linearly arranged (vs. randomly arranged in *C. adangrawi*, *C. chanardi*, *C. huaseesom*, *C. omari*, *C. roticanai*, *C. siamensis*, *C. vandeventeri*). This species is distinguished from *C. adangrawi* and *C. phangngaensis* by having tubercles on lower flanks (vs. absent in *C. adangrawi* and *C. phangngaensis*). This species is distinguished from *C. siamensis* and C. *thachanaensis* by having more 27–29 lamellae under 4^th^ toe (vs. 24–26 in *C. siamensis* and 24 in C. *thachanaensis*).

*Cnemaspis lineatubercularis* sp. nov. is distinguished from *C. chanardi*, *C. huaseesom*, *C. kamolnorranathi*, *C. omari*, *C. roticanai*, *C. siamensis*, and *C. vandeventeri* by the presence of ventrolateral caudal tubercles anteriorly (vs. lacking in *C. chanardi*, *C. huaseesom*, *C. kamolnorranathi*, *C. omari*, *C. roticanai*, *C. siamensis*, *C. vandeventeri*). This species is distinguished from *C. vandeventeri* by having lateral caudal furrows (vs. lacking in *C. vandeventeri*). This species is distinguished from *C. huaseesom* and *C. punctatonuchalis* by having keeled subcaudal scales (vs. lacking in *C. huaseesom* and *C. punctatonuchalis*). This species is distinguished from *C. chanardi*, *C. huaseesom*, *C. omari*, *C. punctatonuchalis*, *C. roticanai*, and *C. siamensis* by having single median row of keeled subcaudals (vs. lacking in *C. chanardi*, *C. huaseesom*, *C. omari*, *C. punctatonuchalis*, *C. roticanai*, *C. siamensis*). This species is distinguished from *C. chanardi*, *C. punctatonuchalis*, *C. siamensis*, and *C. vandeventeri* by lacking enlarged median subcaudal scales (vs. present in *C. chanardi*, *C. punctatonuchalis*, *C. siamensis*, *C. vandeventeri*). This species is distinguished from *C. adangrawi*, *C. chanardi*, *C. huaseesom*, *C. kamolnorranathi*, *C. omari*, *C. punctatonuchalis*, *C. roticanai*, *C. siamensis*, and *C. vandeventeri* by having caudal tubercles restricted to a single paravertebral row on each side (vs. lacking in *C. adangrawi*, *C. chanardi*, *C. huaseesom*, *C. kamolnorranathi*, *C. omari*, *C. punctatonuchalis*, *C. roticanai*, *C. siamensis*, *C. vandeventeri*).

*Cnemaspis lineatubercularis* sp. nov. is distinguished from *C. thachanaensis* by having one postcloacal tubercle in males (vs. lacking in *C. thachanaensis*). This species is distinguished from *C. huaseesom* by having keeled subtibial scales (vs. smooth in *C. huaseesom*). This species is distinguished from *C. adangrawi*, *C. kamolnorranathi*, *C. punctatonuchalis*, *C. siamensis*, *C. thachanaensis*, and *C. vandeventeri* by having yellow coloration in the subcaudal region (vs. lacking in *C. adangrawi*, *C. kamolnorranathi*, *C. punctatonuchalis*, *C. siamensis*, *C. thachanaensis*, *C. vandeventeri*). This species is distinguished from *C. huaseesom*, *C. phangngaensis*, *C. punctatonuchalis*, and *C. roticanai* by lacking dorsal color pattern sexually dimorphic (vs. having in *C. huaseesom*, *C. phangngaensis*, *C. punctatonuchalis*, *C. roticanai*).

## Discussion

The complex geological history of Thailand created a large number of granitic rocky outcrop ecosystems in southern Thailand (Charusiri 1993; Cobbing et al. 2011). This ecosystem supports high levels of species endemism and species diversity of gekkonid lizards, especially species in the genus *Cnemaspis* (see figure 5 in Grismer et al. 2014). The findings of this study provide new data from a poorly studied area in Nakhon Si Thammarat Province, southern Thailand. The results suggest that additional unexplored regions may still harbor unrecognized species of *Cnemaspis* in Thailand.

A decade ago, only four species of *Cnemaspis* were known from Thailand, including *C. biocellata*, *C. chanthaburiensis*, *C. kumpoli*, and *C. siamensis* (Smith 1925; Taylor 1963; Bauer and Das 1998; Grismer et al. 2008a, b). Grismer et al. (2010) described seven new species of *Cnemaspis* (*C. chanardi*, *C. huaseesom*, *C. kamolnorranathi*, *C. narathiwatensis*, *C. niyomwanae*, *C. punctatonuchalis*, and *C. vandeventeri*) from Thailand. Previously, Grismer et al. (2014) described a new species *C. omari* from Perlis, Malaysia, that is also distributed in adjacent Satun Province, Thailand. Wood et al. (2017) described three additional new species of *Cnemaspis* (*C. lineogularis*, *C. phangngaensis*, and *C. thachanaensis*) from southern Thailand. Most recently, two new insular species of *Cnemaspis* (*C. adangrawi* and *C. tarutaoensis*) were described from Tarutao, Adang and Rawi islands of southern Thailand (Ampai et al. 2019). The discovery and description of *C. lineatubercularis* sp. nov. brings the total number of Thai *Cnemaspis* species to 18, representing one-third (33%) of the 60 named species in Southeast Asia.

The new species is known only from the type locality and likely has a narrow geographic distribution. It is expected to be found in other nearby granitic rocky streams in Kam Lon Subdistrict, Lan Saka District, Nakhon Si Thammarat Province. However, additional surveys for this species are needed to clarify the geographic range of the new species. Our findings agree with those of Grismer et al. (2014) that most species of *Cnemaspis* in Thailand are diurnal, granite-associated, lowland species that lack ocelli (Table 7). Further research and additional field surveys in unexplored regions of lowland forest in southern Thailand are needed to better understand the taxonomy, ecology, distribution, biogeography, and conservation of *Cnemaspis* in the region.

## Supplementary Material

XML Treatment for Cnemaspis lineatubercularis

## Appendix 1

List of comparative specimens examined.

*Cnemaspis adangrawi*: Thailand, Satun Province, Mueang Satun District, Adang Island: ZMKU R 00767 (male holotype), ZMKU R 00769–70, THNHM 28206–09 (6 males), ZMKU R 00768, ZMKU R 00771 (2 females); Thailand, Satun Province, Mueang Satun District, Rawi Island: ZMKU R 00773, ZMKU R 00775, THNHM 28210 (3 adult males), ZMKU R 00774, THNHM 28211 (2 females).

*Cnemaspis chanardi*: Thailand, Trang Province, Nayong District, Ban Chong: THNHM 06983 (male holotype); Krabi Province, Klong Thom District: THNHM 012439−40 (males); Mueang Krabi District: THNHM 012436−37 (males), THNHM 012438 (female); Nakhon Si Thammarat Province, Tha Sala District: THNHM 020992 (male); Lansaka district: THNHM 014111 (immature male); Noppitam district: THNHM 013838 (male), THNHM 010705 (male); Surat Thani Province, Ang Thong Island, Mueang Surat Thani District: THNHM 016074 (female).

*Cnemaspis huaseesom*: Thailand, Kanchanaburi Province, Sai Yok District, Sai Yok National Park: THNHM 15909 (male holotype).

*Cnemaspis niyomwanae*: Thailand, Trang Province, Palean District, Thum Khao Ting: THNHM 15909 (female holotype).

*Cnemaspis punctatonuchalis*: Thailand, Prachuap Khiri Khan Province, Thap Sakae District, Huay Yang National Park: THNHM 02001 (male holotype)

*Cnemaspis siamensis*: Thailand, Nakhon Si Thammarat Province, Lan saka District: THNHM 013828 (male); Tha Sala District: THNHM 018265 (male); Phetchabun Province, Nam Nao District: THNHM 01336 (female), THNHM 01337 (male); Phetchaburi Province, Cha-am District: THNHM 01448 (male), THNHM 01449 (immature male); Chumpon Province, Mueang Chumpon District: THNHM 0372 (male); Phato District: THNHM 01086 (male); Surat Thani Province, Vibhawadee District: THNHM 01084 (female); Ang Thong Island, Mueang Surat Thani District: THNHM 015624 (female).

*Cnemaspis vandeventeri*: Thailand, Ranong Province, Kapur District, Klong Naka: THNHM 08261 (male holotype), THNHM 08260 (female).
